# Commentary: Pharmacological Validation of ASIC1a as a Druggable Target for Neuroprotection in Cerebral Ischemia Using an Intravenously Available Small Molecule Inhibitor

**DOI:** 10.3389/fphar.2022.938748

**Published:** 2022-07-05

**Authors:** Shreya Majagi, Sharan Mangat, Xiang-Ping Chu

**Affiliations:** Department of Biomedical Sciences, School of Medicine, University of Missouri-Kansas City, Kansas City, MO, United States

**Keywords:** acid-sensing ion channels, acidosis, ischemic brain injury, neuroprotection, small molecule, C5b

## Introduction

Stroke is a leading cause of death and disability worldwide (Katan and Luft, 2018). Recent studies have shown that emergency interventional treatment of acute ischemic stroke can significantly reduce stroke-related morbidity and mortality (Herpich and Rincon, 2020). Tissue acidosis has been shown to be a consequence of an ischemic stroke (Tóth et al., 2020a; Tóth OM. et al., 2020). An important contender for sensing acidosis is acid-sensing ion channels (ASICs) (Waldmann et al., 1997; Chen et al., 1998; Bässler et al., 2001; Wemmie et al., 2002). ASIC1a is probably the most important ASIC subunit in the brain due to its high expression and sensitivity to pH changes (Waldmann et al., 1997; Xiong et al., 2004; Gründer and Chen, 2010; Faraci et al., 2019; Stark et al., 2019). Specifically, ASIC1a is known to mediate calcium permeability (Waldmann et al., 1997) and has been shown to be an important target of ischemia-induced brain damage (Xiong et al., 2004). As of late, there has been a focus on the potential of therapeutic agents that can target and block ASIC1a subunits with the intention of decreasing ischemic brain injury (Wang et al., 2015; Dibas et al., 2018; Qiang et al., 2018; Wang et al., 2020; Heusser and Pless, 2021). Potential therapeutic agents have been studied including spider venom toxin psalmotoxin (PcTx1) (Xiong et al., 2004; Pignataro et al., 2007; McCarthy et al., 2015; Cristofori-Armstrong and Rash, 2017; Stark et al., 2019) and small molecule inhibitors like amiloride (Leng and Xiong, 2013; Vullo and Kellenberger, 2020). The PcTx1 toxin is formed by purifying spider venom from *Psalmopoeus cambridgei* (Escoubas et al., 2000) and has been shown to be highly selective and effective at inhibiting ASIC1a-containing channels (Escoubas et al., 2003; Chen et al., 2005; Saez et al., 2011). Further, the pure peptide PcTx1 has been used and proved to be effective in several models of neuroprotection (Koehn et al., 2016; Dibas et al., 2018). However, these drugs have some limitations, for example, PcTx1 does not cross the blood-brain barrier (BBB) (Dibas et al., 2018). Consequently, there is a critical need for a therapy that is efficacious and specific to restricting ischemic brain damage in a timely and effective manner.

## Toxin Inspired Compound 5b Inhibits ASIC1a

A recent study was reported in the Frontiers in Pharmacology from Dr. Xu’s laboratory (Qi et al., 2022). They examined a potential therapeutic agent, toxin inspired compound 5b (C5b) that selectively inhibited ASIC1a subunits and ASIC1a-containing channels within ASICs, appearing to reduce ischemia-induced neuronal death in the brain. The effect of C5b on other target than ASICs has never been tested. It has been previously reported that the PcTx1-inspired compound C5b inhibited fast ASIC3-like, but not slow ASIC2-like currents in dorsal root ganglion (DRG) neurons (Buta et al., 2015). The C5b compound is less specific than PcTx1, because it also inhibits ASIC currents recorded from mouse ASIC1a/2a heterotrimers and rat ASIC3 in DRG neurons (Buta et al., 2015). In the present study, Qi et al. focused on if C5b could target ASIC1a and ASIC1a-containing channels in the brain efficaciously. They first examined the selectivity and potency of C5b on different ASIC subunits expressed in CHO cells. They found that C5b at a concentration of 100 nM selectively inhibited ASIC currents recorded from homomeric ASIC1a, heterometric ASIC1a/2a and ASIC1a/2b, but not homomeric ASIC2a and heteromeric ASIC2a/2b channels. Thus, C5b shows a clear selectivity for ASIC1a-containing channels within ASICs. Next, cultured primary cortical neurons were subjected to ischemic conditions, leading to acidosis and cell death. Administering 10 μM C5b to these cells showed to significantly prevent cell death, demonstrating that C5b can alleviate the acid-induced cell death *in vitro*. To test involvement of ASIC1a, cultured cortical neurons from ASIC1a wild-type (WT) and knock-out (KO) mice were used. They found that C5b inhibited acid-induced cell death in cultured cells from ASIC1a-WT but not ASIC1a-KO mice, indicating that C5b′s neuroprotection is through inhibition of the ASIC1a subunit in an *in vitro* cell culture model. It is well known that the ability of pharmacological agents to cross the BBB is critical to the effect of the drug (Khawli and Prabhu, 2013; Peterson et al., 2019). Since the ability to cross BBB is the key to successful drug efficiency, they further examined the pharmacokinetics of C5b after intravenous administration and found that C5b quickly diffused into tissues and after a transitory peak concentration, maintained a relatively constant concentration in the brain during the first 24 h. They then tested the neuroprotective effect of C5b by inducing transient middle cerebral artery occlusion (MCAO) in WT mice. They found that C5b significantly reduced infarct volume and improved the behavioral function in WT mice of this model. Further, they examined the C5b on ASIC1a-KO mice following MCAO and found that C5b did not reveal protection. The data suggested that C5b can cross BBB and exert neuroprotection by inhibiting brain ASIC1a-containing channels (Qi et al., 2022).

## Discussion

Antagonists of ASIC1a have been studied for their protection against ischemic damage (Xiong et al., 2004; Chassagnon et al., 2017; Redd et al., 2021). This study sheds new light on C5b as a novel and small molecule agent for translational stroke research. Inhibitors of ASIC1a have been studied for years in hopes of creating pharmacologic therapeutic agents to treat ischemic strokes (Xiong et al., 2004; Chassagnon et al., 2017). Because of the critical need for a successful agent that blocked ASICs, inhibitors of ASIC1a like amiloride and PcTx1 were studied preclinically (Diochot et al., 2007; Leng and Xiong, 2013; Cristofori-Armstrong and Rash, 2017). For successful treatment of ischemic neuronal damage, systemic administration of the ASIC1a inhibitor is critical. The actuality of implementing current studied ASIC1a blockers has limitations. Amiloride, a small molecule inhibitor of ASIC1a has shown to have poor selectivity (Leng and Xiong, 2013; Dibas et al., 2019). Another well-studied ASIC1a inhibitor is the spider toxin PcTx1 (Escoubas et al., 2000; Escoubas et al., 2003). It has been shown to have difficulty drug delivery (Dibas et al., 2018). The potent inhibitory efficacy of C5b against ASIC1a and ASIC1a-containing channels significantly increased under mild acidosis rather than more severe acidosis (Qi et al., 2022), making it likely to be more effective in the penumbra region when compared to the ischemic core. This could be vital to saving vulnerable brain tissue, reducing the infarct volume, and maintaining a higher level of neurological function in patients. The BBB permeability of C5b, demonstrated by the success of intravenous administration, exemplifies the accessibility of drug administration *in vivo*. Furthermore, C5b was found to target both homomeric ASIC1a and heteromeric ASIC1a-containing channels (Qi et al., 2022). The wider range of use that C5b seems to offer could make it effective in more broad-spectrum application on ASIC-related disorders in the central nervous system. The small molecule nature of C5b could make it useful as a potential therapeutic treatment after strokes due to its permeability of BBB. It is important to note that C5b is administered via a systemic intravenous injection, which first affects peripheral organs at a higher concentration, and then passes the BBB to reach the areas where a pH change has occurred. However, due to C5b’s systemic administration and lower specificity than PcTx1, it is necessary to conduct future work on understanding C5b’s potential side effects at peripheral neurons and organs. It is known there are many ASICs in the peripheral nervous system (PNS) that modulate cutaneous pain (Dibas et al., 2019). Studies on nonselective ASIC inhibitors, like amiloride, have shown that topical administration produces an analgesic effect on postoperative pain in rodents (Dibas et al., 2019). C5b is similarly nonselective due to its inhibition of ASIC3 in DRG neurons (Buta et al., 2015), so it could have effects on pain throughout the entire body. *Anthopleura elagantissima* toxin 2 (APETx2) inhibits ASIC3 and ASIC3-containing channels in afferent bone sensory neurons and has been used to treat inflammatory bone pain (Morgan et al., 2020). By looking at drugs with similar inhibitory properties as C5b, there is a possibility that C5b could produce effects on various systems, and this will require further study. Particularly, C5b’s effects on the ample ASIC1b channels of the PNS has not been thoroughly studied. These ASIC1b channels are involved in persistent pain (Verkest et al., 2021), so if C5b can inhibit them, there is the potential for analgesic use. Examining potential adverse side effects of C5b is necessary, as well as testing for the ideal dosage and timing of administration. In a clinical setting, the current goal for ischemic stroke patients is to administer tissue plasminogen activator (tPA) within 4.5 h (Cheng and Kim, 2015). Unfortunately, many stroke patients are unable to make it to the hospital and receive tPA treatment in this short-time window. In order to account for this clinical obstacle in stroke treatment, future studies are needed to assess the efficacy of C5b at various time increments after ischemic injury, following the model of a similar study done for PcTx1 (Pignataro et al., 2007). Therefore, the time window of neuroprotection by C5b would help determine a potential therapeutic window for drug administration, which could be compared to tPA’s window of 4.5 h. It would also be useful to look at which drugs most stroke patients are currently taking at the time of ischemic injury, and how these could interact with C5b’s administration and effects. Lastly, it is also critical to examine whether C5b affects the function of any other ion channels or receptors other than ASICs.
